# Polymer Lasing in a Periodic-Random Compound Cavity

**DOI:** 10.3390/polym10111194

**Published:** 2018-10-26

**Authors:** Tianrui Zhai, Xiaofeng Wu, Songtao Li, Shuyan Liang, Lianze Niu, Meng Wang, Shengfei Feng, Hongmei Liu, Xinping Zhang

**Affiliations:** 1 Institute of Information Photonics Technology and College of Applied Sciences, Beijing University of Technology, Beijing 100124, China; wuxiaofeng0108@163.com (X.W.); songtaoli2001@126.com (S.L.); 17110720020@fudan.edu.cn (S.L.); niulianze@126.com (L.N.); wangmeng004@emails.bjut.edu.cn (M.W.); hmliu@bjut.edu.cn (H.L.); 2 Department of Physics, Capital Normal University, Beijing 100048, China; fengshengfei@hotmail.com

**Keywords:** distributed feedback laser, random laser, polymer laser, compound cavity

## Abstract

Simultaneous distributed feedback (DFB) lasing and linear polarized random lasing are observed in a compound cavity, which consists of a grating cavity and a random cavity. The grating cavity is fabricated by interference lithography. A light-emitting polymer doped with silver nanoparticles is spin-coated on the grating, forming a random cavity. DFB lasing and random lasing occur when the periodic-random compound cavity is optically pumped. The directionality and polarization of the random laser are modified by the grating structure. These results can potentially be used to design integrated laser sources.

## 1. Introduction

Polymer lasers have attracted broad attention in recent years due to the additional degree of freedom they afford in the development of versatile light sources [1,2,3,4,5,6]. Multi-wavelength, distributed-feedback (DFB) polymer lasers are achieved in various cavities, including cascaded cavities [7,8,9,10], arrays [11,12], and compound cavities [13,14,15,16]. Generally speaking, scattering is detrimental to laser action because it induces loss to the DFB polymer lasers. However, the combination of multiple scattering and high gain leads to random laser action [17,18,19,20,21,22]. So, simultaneous DFB lasing and random lasing are expected in one single integrated device if the scattering particles are introduced appropriately into the DFB cavity, which forms a periodic-random compound cavity. The thickness of the integrated device is much thinner than the total thickness of the two components. In addition, the introduction of randomness to a periodic structure enhances light localization. It is well known that the scattering loss is pretty large and the threshold of the random laser is usually very high. Cao et al. has reported that with a proper increase in the degree of disorder, the lasing threshold in such a cavity decreases [23]. So, introducing some disorder to the period structure helps to lower the laser threshold.

In this work, a periodic-random compound cavity is proposed to achieve DFB lasing and random lasing simultaneously. The compound cavity consists of a grating structure and a polymer layer embedded with silver nanoparticles (Ag NPs). The grating is fabricated using interference lithography. Then, the polymer solution with Ag NPs is spin-coated onto the grating, forming an active waveguide. Upon optical pumping, directional DFB lasing and non-directional random lasing are observed simultaneously. The angular distribution and polarization of the output of the random laser are modified by the grating structure. The polarization state of the random laser can be changed from non-polarization to linear polarization. Furthermore, by adjusting the period of the grating, mode competition occurs when the DFB lasing peak overlaps the random lasing peak.

## 2. Fabrication of the Periodic-Random Compound Cavity

**Figure 1:**
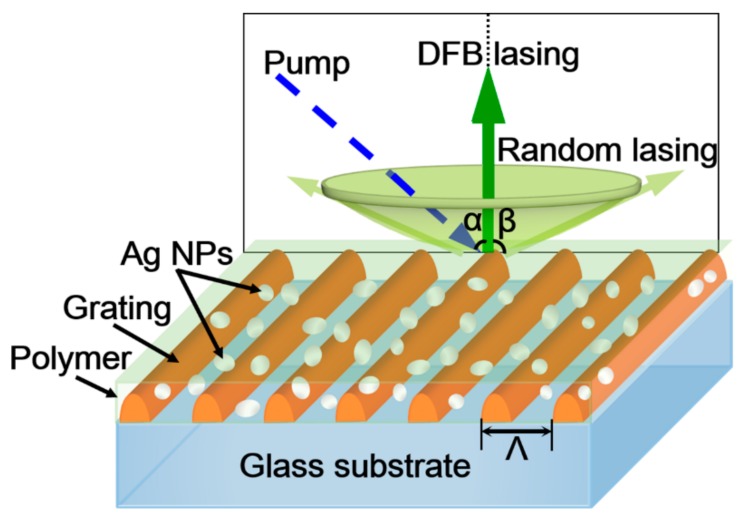
Schematic of a polymer laser based on the periodic-random compound cavity. The blue/green arrow denotes the direction of the pump/DFB lasing. The light-green cone indicates the output direction of the random laser. α, β are the incident angle of the pump and the solid angle of the random laser output, respectively. α = 20°. Λ is the period of the grating.

In the experiment, the photoresist (PR, AR-P 3170, Allresist, Strausberg, Germany) was spin-coated onto a glass substrate (15 × 15 × 1 mm) with a speed of 2000 rpm, forming a film of 150-nm thickness. A short-pulsed diode-pumped solid-state laser (343 nm, 1 ns, 2 kHz, 100 μJ) was employed as the ultraviolet light source. The laser beam was split into two equal parts, which were then overlapped on the surface of the PR film for 5 s. Then the PR film was developed in a sodium hydroxide solution for 3 s. The fabrication process has been reported in our previous paper [24]. So, an interference pattern can be recorded on the PR film, which acts as the DFB cavity in Figure 1. The period of the pattern (Λ) is determined by the included angle (θ) between the two interference beams, which follows the formula Λ = λ⁄2sinθ, where λ is the wavelength of the laser. The Ag NPs are synthesized using a one-step method [25,26]. The ligand attached to the Ag NPs is *n*-butylamine. The mean diameter of the Ag NPs is around 70 nm [27]. The Ag NPs are solved in xylene, forming an ink of Ag NPs with a concentration of 4 mg/mL. A light-emitting polymer, poly[(9,9-dioctylfluorenyl-2,7-diyl)-alt-*co*-(1,4-benzo-(2,1′,3)-thiadiazole)] (F8BT, American Dye Source), is used as the active material. The ink of Ag NPs and the F8BT solution were mixed with the volume ratio of 1:1, forming the mixsolution. Then, the mixed solution was spin-coated on the PR grating with a speed of 1500 rpm. Thus, the compound cavity with a thickness about of 230 nm is constructed by PR gratings, polymer film, and Ag NPs. The Ag NPs are randomly distributed in the polymer film, which acts as the random cavity in Figure 1. The thicknesses of the films are measured using a stylus profiler (Nanomap-500LS, AEP Technology, Santa Clara, CA, USA).

Figure 1 illustrates the schematic diagram of the polymer laser based on the periodic-random compound cavity, which is similar to the waveguide grating structure [24]. The green arrow denotes the output direction of the DFB laser, which is a surface-emitting device. The light-green cone indicates the non-directional output of the random laser.

**Figure 2:**
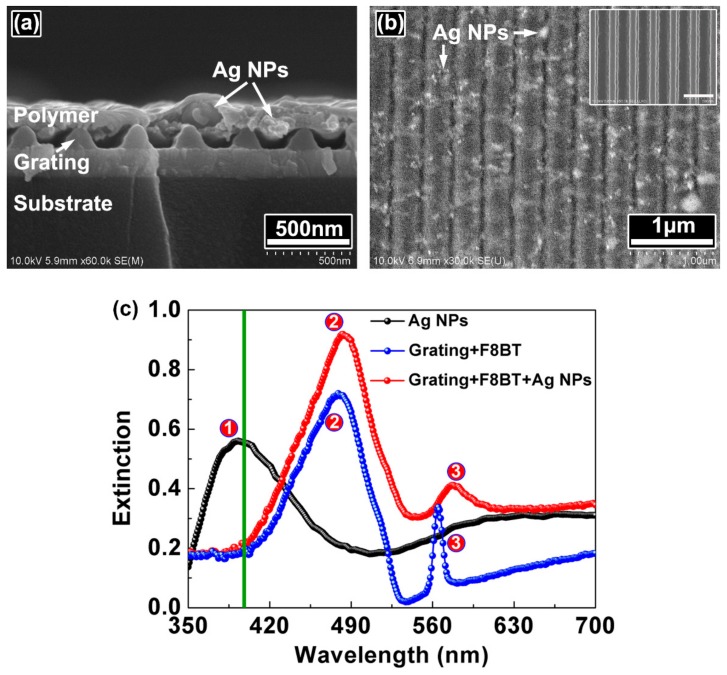
SEM images of (**a**) the cross section and (**b**) the top view of the periodic-random compound cavity. The inset in (**b**) shows the SEM image of the grating without a polymer cladding. The scale bar is 500 nm. (**c**) Extinction spectra of Ag NPs (black filled circles), the grating coated with polymer (blue filled circles), and the grating coated with polymer and Ag NPs (red filled circles). The green line indicates the wavelength of the pump light. Symbols ①, ②, and ③ indicate the corresponding extinction peaks.

The morphologies of the periodic-random compound cavity are measured by scanning electron microscopy (SEM, Hitachi S-4800, Hitachi, Tokyo, Japan). Figure 2 shows the SEM images of the cross section and the top view of the compound cavity. Ag NPs can be observed clearly in the polymer film, which provides scattering to stimulate spontaneous emission into random laser emission.

## 3. Spectra Characterization of the Periodic-Random Compound Cavity

The inset in Figure 2b demonstrates the grating structure before spin-coating the polymer layer, which is a periodic cavity. The period of the grating is 350 nm. Figure 2c presents the extinction spectra of the Ag NPs (black filled circles) on the substrate, the grating coated with polymer (blue filled circles), and the grating coated with polymer and Ag NPs (red filled circles). The plasmonic resonance peak of the Ag NPs on the substrate locates at 393 nm, which shifts towards the longer wavelength when the Ag NPs are doped into the polymer. The radiation of the polymer molecules is scattered by the Ag NPs due to the localized surface plasmon resonance of Ag NPs. Part of the scattered light is reflected back at the polymer/air interface to propagate in the polymer film and diffracted by the grating structure. The extinction peak of F8BT is at 480 nm, as denoted by the symbol ②. The spectral peaks located at about 565 nm are attributed to the waveguide resonance mode of the waveguide grating structure [24]. Note that the plasmonic resonance of Ag NPs is weak near the waveguide resonance peak, which indicates the relatively weak influence of the scattering of the Ag NPs on the operation of the DFB laser.

**Figure 3:**
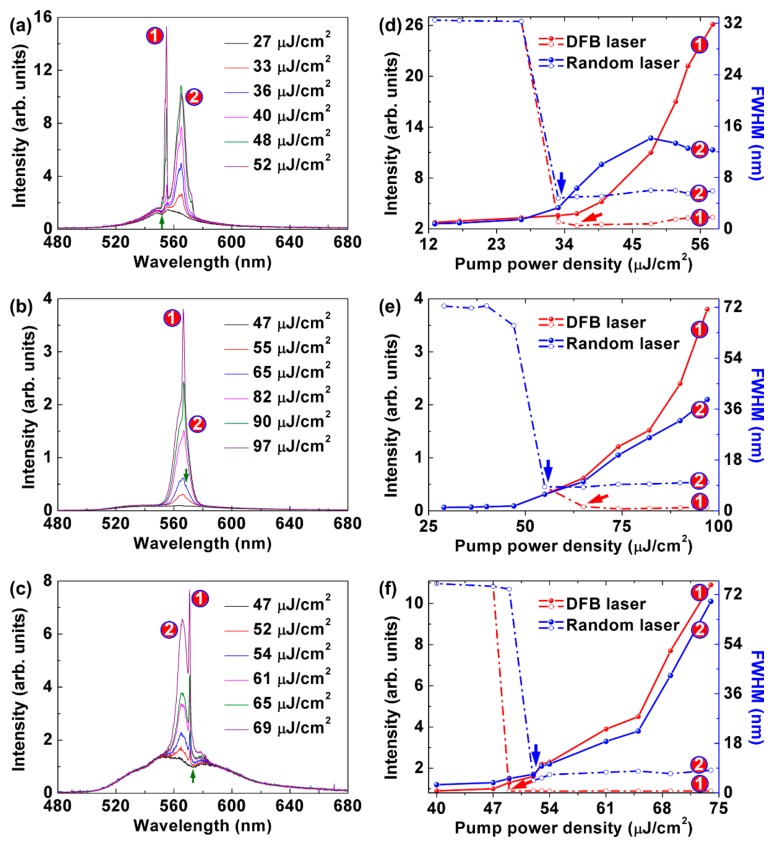
Emission spectra of the compound polymer lasers measured at different pump fluences. The periods of the gratings are (**a**) 350 nm, (**b**) 360 nm, and (**c**) 370 nm, respectively. FWHM and output intensity of the compound polymer lasers with (**d**) 350 nm, (**e**) 360 nm, and (**f**) 370 nm grating structures as a function of the pump fluence. The thresholds of the DFB lasing and random lasing are indicated by the red and the blue arrows, respectively.

During the spectral measurements, the periodic-random compound cavity is pumped by a 200-fs laser. The wavelength of the pump light is 400 nm, as indicated by the green line in Figure 2c. The pulse repetition frequency is 1 kHz. The pump power is varied from 0 to 40 mW by a neutral optical attenuator. The diameter of the pump beam is 2 mm. The laser output is measured perpendicular to the sample surface with an optical spectrometer (Maya 2000 Pro, Ocean Optics, Largo, FL, USA). Figure 3 shows the emission spectra of the compound polymer lasers.

The random laser emission is centered at 565 nm, and has a linewidth less than 10 nm at full width at half maximum (FWHM) in Figure 3a–c. The emission wavelength of the DFB polymer laser can be tuned by changing the period of the grating [28]. The wavelengths of the DFB lasing are 555, 566, and 571 nm for the 350, 360, and 370 nm cavity, respectively, as shown in Figure 3a–c. The FWHM and output intensity as a function of the pump fluence are shown in Figure 3d–f. The FWHM of the DFB lasing is about 1 nm. The thresholds of the 350, 360, and 370 nm cavities are 34, 54, and 49 μJ/cm^2^, respectively. It can be seen that the thresholds of the random lasing are 33, 55, and 52 μJ/cm^2^ in Figure 3d–f, respectively. Note that the separation of the two lasing peaks are 10, 1, and 6 nm in Figure 3a–c, respectively. So, the thresholds of the DFB lasing and the random lasing decrease with increasing the separation of the two lasing peaks, which is attributed to the mode competition.

For the surface-emitting DFB laser in this work, the spectra in Figure 3a–c show the clear dips in emission at 552, 568, and 573 nm, respectively, as indicated by the green arrows. The dips appear in the spectra because of the presence of the photonic band gap at the Bragg condition. The DFB lasing originates from the dip, which can be regard as a band-edge lasing action [29]. The effective index of the waveguide mode is decided by $n_{eff}=\lambda /\Lambda$, where λ is the DFB lasing wavelength and Λ is the period of the grating structure. So, the effective refractive indices of the 552, 568, and 573 nm modes are calculated as 1.58, 1.58, and 1.55, respectively.

**Figure 4:**
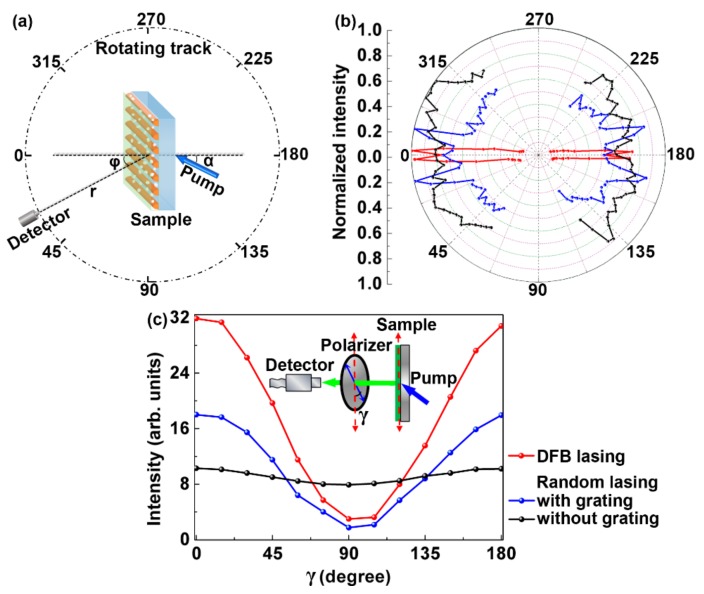
(**a**) Schematic of the emission spectra measurement at different angles (φ). α = 20°. r = 30 mm. (**b**) Measured angular emission spectra of the DFB lasing (the red curve) and the random lasing (the blue curve) in a compound cavity. The black curve shows the angular spectra of the random laser without grating. All spectra were measured within 60 degrees (φ < 60°). (**c**) The polarization state of the random laser with (the red curve) and without (the black curve) the grating. The inset shows the measurement setup. γ is the angle between the grating groove direction (shown by the red arrow) and the polarization direction of the polarizer (shown by the blue arrow). The polarization state of the DFB laser is indicated by the blue curve.

To characterize the directionality of the laser output, the emission spectra are measured as a function of the angle (φ), as shown in Figure 4a. φ is the included angle of the normal of the sample surface and the axis of the detector. The detector is mounted on a rotating stage kept at a distance of 30 mm from the center of the sample. The diameter of the detector is 600 μm. So, the opening angle of the detector is about 1.15°. Figure 4b presents the experimental angular emission spectra of the compound polymer laser. It is difficult to perform a large-angle measurement due to the alignment of the small-diameter detector (600 μm). So, all emission spectra were measured within 60 degrees (φ < 60°). The angular spectra of the DFB lasing and the random lasing are indicated by the red and the blue curves, respectively. Four peaks are observed in the angular spectra of the random lasing. The diffraction profile of the random laser follow the characteristic relation: $sin\theta =\left(mk_{G}-k_{mode}\right)/k_{0}$ [30], where m is the order of scattering (m=1 for the first order scatterings), $k_{G}$ is the grating vector, $k_{mode}$ is a waveguide mode, $k_{0}$ is the wavenumber. The angular spectrum of the random laser without grating is presented as a comparison (the black curve) in Figure 4b. It can be seen that the directionality of the random lasing is modified by the grating structure.

The polarization state of the output of the compound device is measured carefully. During the measurements, a rotatable polarizer is placed between the laser device and the spectrometer. γ is the angle between the grating groove direction and the polarization direction of the polarizer, and is changed from 0° to 180° with a step of 15°. The optical spectrometer is employed to collect the emitting spectra to measure the polarization state of the laser output. As a comparison, the polarization state of the random laser without the grating is also measured as denoted by the black curve in Figure 4c. Note that the polarization ratios between parallel (γ = 0°) and perpendicular (γ = 90°) polarizations are 10.2 and 1.3 for the random laser with and without the grating, respectively. The polarization ratio of the DFB lasing is 10.6, which is a linear polarization oriented along the grating groove direction. So, the polarization state of the random laser changes from non-polarization to linear polarization due to the modification of the grating structure.

The polarization dependency of the compound device is well explained by the slab waveguide theory. In this work, the polymer waveguide is so thin that only the first transverse electric waveguide mode (TE_0_) is supported [31]. The polarization of the TE_0_ mode is parallel to the grating groove direction, which influences the polarization of the laser output due to the Bragg scattering.

## 4. Conclusions

In conclusion, a periodic-random compound structure is used as a laser cavity, which consists of a grating structure and a polymer film embedded with Ag NPs. The grating structure acts as the periodic cavity. The Ag NPs act as the random cavity. DFB lasing and random lasing are observed simultaneously in the compound cavity under optically pumped conditions. The threshold of the laser device changes with the separation of the two peaks of the DFB lasing and random lasing due to the mode competition. The directionality and polarization of the random lasing is modified by the periodic cavity. Linear polarized random laser can be achieved by the proposed method. These results provide a simple method for designing compact laser sources.
